# 82. Incidence rates of invasive aspergillosis among lung transplant recipients in timeperiods with universal and targeted antifungal prophylaxis - a nationwide cohort study

**DOI:** 10.1093/ofid/ofac492.007

**Published:** 2022-12-15

**Authors:** Cornelia G Crone, Signe Marie Wulff, Bruno Ledergerber, Pia Bredahl, Jannik Helweg-Larsen, Maiken C Arendrup, Michael Perch, Marie Helleberg

**Affiliations:** Rigshospitalet, Copenhagen University Hospital, Copehagen N, Hovedstaden, Denmark; CHIP -Centre of Excellence for Health, Immunity and Infections, Rigshospitalet, Copenhagen, Hovedstaden, Denmark; Rigshospitalet, University of Copenhagen, Rorbas, Zurich, Switzerland; Rigshospitalet, Copenhagen, Hovedstaden, Denmark; Rigshospitalet, Copenhagen University Hospital, Copehagen N, Hovedstaden, Denmark; Statens Serum Institut, Copenhagen, Hovedstaden, Denmark; University of Copenhagen, Copenhagen, Hovedstaden, Denmark; Rigshospitalet, Copenhagen University Hospital, Copehagen N, Hovedstaden, Denmark

## Abstract

**Background:**

The best stategy for prevention of invasive aspergillosis (IA) after lung transplantation (LTX) has not been determined. In 2016, Danish guidelines for IA prophylaxis was changed from 12 weeks of universal prophylaxis with voriconazole to targeted prophylaxis with posaconazole and inhaled amphotericin B for high risk patients. In a previous study we found significantly higher rates of adverse events in the period with universal prophylaxis. The objectives of this study were to compare rates of IA 1) in time periods before and after change of guidelines; 2) during persontime on vs. persontime without antifungal prophylaxis in the total study period.

**Methods:**

In a Danish nationwide study we included all adult lung transplant recipients (LTXr), 2010–2019. Proven and probable IA were defined using ISHLT criteria. Patients were censored at the first of following events: death, retransplantation, end of 2019, or one year after LTX. The cumulative hazard of IA was calculated using the Nelson Aalen estimator. Rates of IA were analyzed in bi- and multivariate Poisson regression models adjusted for the time period after LTX (0–6 and 6–12 months). Persontime on protocol prophylaxis (start to end + 14 days) was treated as time-updated variable.

**Results:**

A total of 295 LTXr were followed for 245 person-years, of whom 183/193 vs. 6/102 initiated antifungal prophylaxis during the universal vs. the targeted prophylaxis period (Table 1). IA was diagnosed in 34/193 (17.6%) vs. 15/102 (14.7%) LTXr the first year after transplantation in the universal vs. targeted prophylaxis period. Overall, similar cumulative hazards of IA were observed between the universal and targeted prophylaxis period (p=0.59, Figure 1). The adjusted incidence rate ratio (aIRR) of IA during the universal vs. targeted prophylaxis period was 1.30 (95% CI 0.69–2.44), (Table 2). In the total study period, the aIRR of IA during persontime on vs. persontime without antifungal prophylaxis was 0.87 (95% CI 0.40–1.88), (Table 2).

Characteristics of lung transplant recipients in time periods with universal voriconazole and targeted posaconazole and inhaled amphotericin B prophylaxis.

Proven and probable invasive aspergillosis including anastomosis infection, trakeobronkitis and pneumonia according to ISHLT criteria. *) Underlying diseases categorized as high risk of invasive aspergillosis.

Incidence rates (IR) and incidence rate ratios (IRR) of invasive aspergillosis in lung transplant recipients in two multivariate models.

Poisson regression models comparing rates of IA in model 1) during universal prophylaxis time period versus targeted prophylaxis time period and in model 2) during persontime on prophylaxis (start to end + 14 days) versus persontime without prophylaxis in the total study period 2010–2019.

Adjusted for time after LTX IRR: Bivariable model including the listed variables in bold and adjustment for time after transplantation (time-updated). Fully adjusted IRR: Multivariable model with adjustment for sex, age, high risk underlying disease, single/double lung transplant, Aspergillus prior to transplantation and time after transplantation (time-updated).

Abbreviations: LTX = lung transplant recipients; CI = confidence interval; IR = incidence rates per 100 person-years; IRR = incidence rate ratio; Ref. = reference.

Cumulative hazards of invasive aspergillosis the first year after lung transplantation in time periods with universal voriconazole versus targeted posaconazole and inhaled amphotericin B prophylaxis.

**Conclusion:**

A strategy of targeted prophylaxis, for high risk patients only, may be preferred over universal voriconazole prophylaxis after LTX due to similar rates of IA and lower rates of adverse events.

**Disclosures:**

**Maiken C. Arendrup, DMSci, PhD, MD**, Chiesi, Gilead: Honoraria|F2G, Cidara, Scynexis: Grant/Research Support **Michael Perch, MD**, Boeringer-Ingelheim: Travel grant|PulmonX: Expert Testimony|Roche: Grant/Research Support|Takeda: Expert Testimony|Therakos: Honoraria|Zambon: Advisor/Consultant|Zambon: Expert Testimony **Marie Helleberg, PhD, DMSc**, AstraZeneca: Advisor/Consultant|Gilead: Advisor/Consultant|Gilead: Honoraria|GSK: Advisor/Consultant|GSK: Honoraria|Janssen: Advisor/Consultant|Roche: Advisor/Consultant|Sobi: Advisor/Consultant.

